# Role of L-Particles during Herpes Simplex Virus Infection

**DOI:** 10.3389/fmicb.2017.02565

**Published:** 2017-12-19

**Authors:** Christiane S. Heilingloh, Adalbert Krawczyk

**Affiliations:** ^1^Department of Immunomodulation, University Hospital Erlangen, Erlangen, Germany; ^2^Institute for Virology, University Hospital Essen, University of Duisburg-Essen, Essen, Germany

**Keywords:** Herpes simplex virus, L-particles, H-particles, virus maturation, immune evasion

## Abstract

Infection of eukaryotic cells with α-herpesviruses results in the formation and secretion of infectious heavy particles (virions; H-particles) and non-infectious light particles (L-particles). Herpes simplex virus type 1 (HSV-1) H-particles consist of a genome-containing capsid surrounded by tegument proteins and a glycoprotein-rich lipid bilayer. Non-infectious L-particles are composed mainly of envelope and tegument proteins and are devoid of capsids and viral DNA. L-particles were first described in the early nineties and from then on investigated for their formation and role during virus infection. The development and secretion of L-particles occur simultaneously to the assembly of complete viral particles. HSV-1 L-particles are assembled by budding of condensed tegument into Golgi-delivered vesicles and are capable of delivering their functional content to non-infected cells. Thereby, HSV-1 L-particles contribute to viral pathogenesis within the infected host by enhancing virion infectivity and providing immune evasion functions. In this review we discuss the emergence of HSV-1 L-particles during virus replication and their biological functions described thus far.

## Introduction

Eukaryotic cells secrete a wide spectrum of membrane-enclosed vesicles containing proteins, RNA, microRNA or DNA (D’Souza-Schorey and Clancy, 2012). These vesicles are commonly termed extracellular vesicles (EVs) and comprised of microvesicles, apoptotic bodies, and exosomes (Kalamvoki and Deschamps, 2016). Overall, microvesicles play an important role in intercellular communication, which is essential for the coordination and proper organization of different cell types in multicellular systems (Corrado et al., 2013). Interestingly, microvesicles secreted by virus-infected cells contain viral material and influence the course of infection by facilitating viral replication and/or activating immune evasion strategies (Meckes and Raab-Traub, 2011). The formation of non-infectious subviral microvesicles and/or virus material containing exosomes was described for a broad range of viruses such as HIV (Mack et al., 2000), HBV (Blumberg, 1977; Hu and Liu, 2017) and all three families of Herpesviruses (Meckes and Raab-Traub, 2011). Cells infected with α-herpesviruses like herpes simplex virus type 1 (HSV-1) or the swine pseudorabies virus (PrV) produce not only infectious virions (H-particles) but also non-infectious microvesicles (L-particles). HSV-1-related L-particles were first separated from infectious virions by Szilagyi and Cunningham (1991) by density gradient centrifugation. Since then numerous studies have been performed to characterize the maturation of L-particles, their physical properties and role in transporting biologically active macromolecules from infected to non-infected cells.

## Structural Features of L-Particles

**FIGURE 1:**
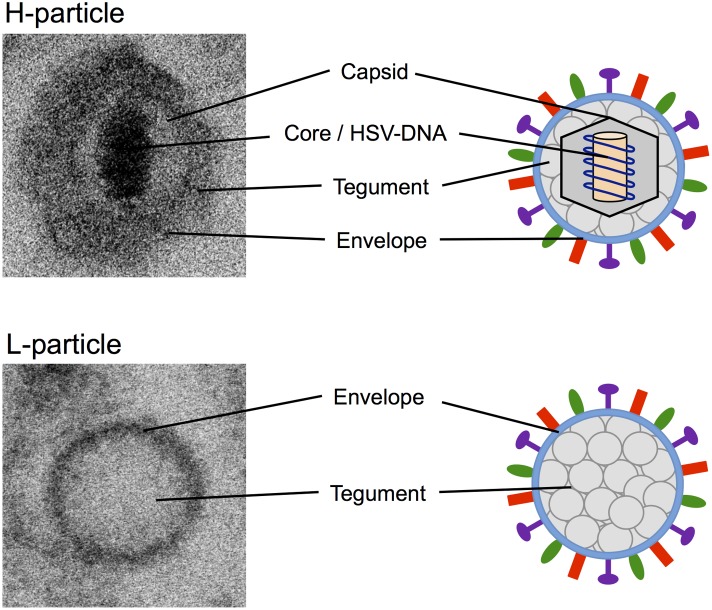
Electron microscopy and schematic pictures of α-herpesviral H- and L-particles. EM pictures of HSV-1 H- and L-particles were taken with the ZEISS FIB-SEM 540 Crossbeam transmission electron microscope.

H-particles of α-herpesviruses are characteristically structured in core, nucleocapsid, tegument, and lipid envelope (**Figure 1**; Pellett and Roizman, 2007). The core, which is enclosed by an icosahedral capsid with a diameter of approximately 100 nm, consists of the viral double stranded DNA genome attached to a fibrillar protein matrix. The viral capsid is embedded in the tegument consisting of more than 15 different viral proteins. Surrounding the capsid and tegument is a lipid bilayer envelope which is important for the attachment to host cells and viral entry, and in which 12 different virally encoded glycoproteins are inserted (Spear and Longnecker, 2003; Akhtar and Shukla, 2009). In contrast, L-particles predominantly consist of a uniformly granular tegument and a lipid glycoprotein-containing envelope and lack any nucleocapsid and viral DNA (Szilagyi and Cunningham, 1991; McLauchlan and Rixon, 1992; Szilagyi and Berriman, 1994). Due to the absence of the viral genome, L-particles are non-infectious. Furthermore, L-particles are similar in size to H-particles but more heterogeneous. With a diameter of approximately 140 nm L-particles are smaller than virions, which have 180 nm on average (Szilagyi and Berriman, 1994; Dargan and Subak-Sharpe, 1997). This heterogeneity of L-particles is most probably caused by the lack of the nucleocapsid, which defines the specific viral shape.

Although, many proteins of the tegument and the viral envelope were common to both types of viral particles, there are some features that are unique to L-particles. Szilagyi and Cunningham (1991) postulated that L-particles include up to five phosphoproteins that were not detected in H-particles. One of these proteins is the immediate early protein ICP4 (infected cell protein 4) that is known as the major transcriptional regulatory protein of HSV-1 (McLauchlan and Rixon, 1992; Lester and DeLuca, 2011). Furthermore, a large proportion of L-particles were found to contain one or more internal membrane-surrounded sphere-like vesicles, called inclusion vesicles (IVs), which are embedded in the tegument (Szilagyi and Berriman, 1994). Besides their variation in size and number, it has been postulated that these IVs contain at least some of the five phosphoproteins unique to L-particles (Szilagyi and Berriman, 1994).

## Maturation of L-Particles

**FIGURE 2:**
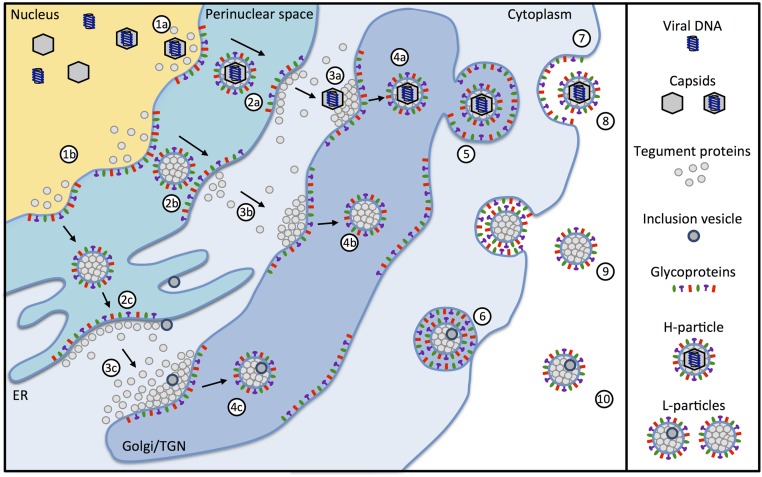
Maturation of α-herpesviral H- and L-particles. Capsids containing viral DNA (1a) are enveloped together with tegument proteins by budding from the inner nuclear membrane resulting in the formation of primary enveloped H-particles (2a). Simultaneously, the budding process can occur with tegument proteins alone in the absence of capsids (1b) resulting in the formation of empty particles within the perinuclear space (2b and 2c). The primary envelope of H-particles fuses with the outer nuclear membrane leading to translocation of the nucleocapsids into the cytosol (3a). L-particles fuse with the outer nuclear membrane (3b) or when migrating through the endoplasmatic reticulum (3c). Nucleocapsids acquire additional tegument proteins and obtain the final envelope by budding into the trans-Golgi network (TGN) (4a). Additionally, L-particles containing tegument proteins (4b) are formed. Some of the L-particles also include inclusion vesicles (IVs) (4c). The origin of IVs can be the endoplasmatic reticulum, membranes of the TGN or other membrane compartments. After secondary envelopment, the H- and L-particles are packed in secretory vesicles (5), transported to the plasma membrane (6) and finally released from the cell (7). The particles released from the infected cell are infectious H-particles (8) and non-infectious L-particles (9), which can additionally contain inclusion vesicles (10).

The complex architecture of herpesviruses, which consists of more than 30 virally encoded proteins, requires a sophisticated production pathway that involves several cellular compartments (Mettenleiter, 2006). After viral gene expression and DNA-synthesis is completed, viral capsid proteins are translocated from the cytoplasm to the nucleus. There, viral capsids assemble and incorporate herpesvirus-DNA. To exit the nucleus, DNA-filled viral capsids use vesicle-mediated transport across the nuclear membrane (**Figure 2**, 1a). Prior studies demonstrated that the viral proteins pU_L_31 and pU_L_34 of HSV-1 and the swine α-herpesvirus PrV form a nuclear egress complex, which is required for budding and vesicle formation at the inner nuclear membrane (Klupp et al., 2007; Mettenleiter et al., 2013). Interestingly, it was shown that pU_L_31 directly interacts with the capsid facilitating the nuclear egress (Funk et al., 2015). In the absence of either protein, the capsids are not primary enveloped and accumulate at the nucleus (Mettenleiter, 2004). Although this interaction is crucial for H-particle formation, there is no evidence that pU_L_31 and pU_L_34 are involved in L-particle formation. Accordingly, a study of Granzow et al. (2001) dealing with the infectious laryngotracheitis virus (ILTV) suggested that L-particles are also formed in the absence of capsids. Small molecules are transported through the nuclear membrane via the nuclear pore complex (Pante and Kann, 2002). Macromolecular protein complexes are unable to pass through the nuclear pore complex and are transported from the nucleus via vesicles into the cytoplasm (Speese et al., 2012). After primary envelopment, capsids and condensed tegument proteins reach the cytoplasm by de-envelopment at the outer nuclear membrane (**Figure 2**, 2a; Smith, 1980; Mettenleiter, 2004). After translocation through the nuclear membrane into the cytoplasm, the non-enveloped α-herpesvirus capsids gain their final tegument via adding further components as well as remodeling existing tegument proteins (**Figure 2**, 3a). Secondary envelopment then occurs by budding into cytoplasmic membranes (**Figure 2**, 4a; Mettenleiter, 2006). These can be the golgi, the *trans*-Golgi network (TGN) or endosomes (Harley et al., 2001; Owen et al., 2015). During infection, similar quantities of H- and L-particles are produced (Szilagyi and Cunningham, 1991). L-particles are formed via the budding of condensed tegument through the inner nuclear membrane into the perinuclear space (**Figure 2**, 2b). Besides the perinuclear space and in contrast to H-particles, L-particles can also be found in the cisternae of the rough endoplasmic reticulum (**Figure 2**, 2c; Granzow et al., 2001; Aleman et al., 2003). Further maturation of L-particles occurs in a way similar to H-particles by the budding of condensed tegument into Golgi-derived vesicles (**Figure 2**, 3b, 3c; Aleman et al., 2003). However, it cannot be excluded that L-particles are also initiated directly in the cytoplasm without the involvement of the nucleus. Interestingly, L-particles can contain IVs. The origin of these vesicles is not known, but is most likely identical to membrane compartments such as the endoplasmatic reticulum or membranes of the TGN (Ibiricu et al., 2013). Since protein complexes are transported via vesicles from the nucleus to the cytoplasm (Speese et al., 2012), one cannot rule out that IVs may also originate from the nuclear membrane. Nevertheless, while the specific role of IVs is not clear, it is suggested that some of the five phosphoproteins which are unique to L-particles are incorporated in these vesicles (Szilagyi and Berriman, 1994).

Viral particles are finally released from the infected cell via exocytosis. After secondary envelopment, the H- and L-particles are packed into secretory vesicles (**Figure 2**, 5) and transported to the plasma membrane (**Figure 2**, 6). There, the vesicles fuse with the cell membrane and release their content out of the cell (**Figure 2**, 7; Foster and Kousoulas, 1999). This ultimately results in mature H-particles (**Figure 2**, 8) as well as L-particles with or without additional IVs (**Figure 2**, 9, 10, respectively; Ibiricu et al., 2013). Besides, cells infected with α-herpesviruses were reported to release empty enveloped capsids (Aleman et al., 2003) and exosomes containing viral material such as mRNAs miRNAs and proteins (Kalamvoki and Deschamps, 2016). Furthermore, observation from a prior study indicated that capsids and IVs could be found in the same virion, suggesting that IVs are not exclusive to L-particles (Ibiricu et al., 2013).

## Role of L-Particles During Infection

L-particles are produced by all α-herpesviruses (e.g., HSV-1/2, PRV, EHV-1, BHV-1, and VZV) tested thus far in various human and animal cells, suggesting that these particles fulfill one or more important functions during infection (McLauchlan and Rixon, 1992; Dargan et al., 1995; Dargan and Subak-Sharpe, 1997; Subak-Sharpe and Dargan, 1998). Moreover, L- and H-particles seem to share the same cellular receptors for cell entry and use the same mechanisms for attachment, fusion and tegument protein release (Dargan and Subak-Sharpe, 1997). Therefore, L-particles are as efficient as H-particles in delivering viral proteins to uninfected cells. Hence, L-particles may play an important role particularly in the early stages of herpesvirus infection (McLauchlan and Rixon, 1992). This suggestion is strengthened by the observation that the virion host shutoff (vhs) protein is incorporated into L-particles and that they can be delivered in a functional and biological active state (McLauchlan and Rixon, 1992; Subak-Sharpe and Dargan, 1998). Since vhs is thought to be involved in mechanisms for viral immune escape via promoting viral gene expression, the transfer of this protein to uninfected bystander cells might lead to the priming of surrounding cells in order to enhance the efficiency of infection (Tigges et al., 1996).

Recent findings support the hypothesis that L-particles may participate in mechanisms of immune evasion. It has been demonstrated that the expression of CD83 on mature dendritic cells, which are professional antigen presenting cells and thereby representing key players for the induction of an effective antiviral immune response, can be down-modulated via L-particles (Heilingloh et al., 2015). Since CD83 is important for effective T-cell priming, this mechanism promotes an evasion of the virus from the host’s immune response (Kruse et al., 2000; Prechtel et al., 2007). Moreover, mature dendritic cells are not permissive for an HSV-1 infection and therefore do not produce new progeny viruses but high amounts of L-particles (Goldwich et al., 2011; Heilingloh et al., 2015). Therefore, the virus has evolved strategies to influence the host’s immune response even in the absence of viral gene expression.

Besides, additional studies from Dargan and Subak-Sharpe (1997) indicated that L-particles might play a role in enhancing viral replication in cases of, e.g., reactivation from latency. Furthermore, they suggested that L-particles may promote infection of otherwise semipermissive cell types or complement the defective function of partially inactivated coinfecting virions. The fact that L-particles contain some viral proteins, which are not incorporated into full virions, suggests that L-particles may have functions that are not shared by H-particles (McLauchlan and Rixon, 1992). Relating to these hypotheses, L-particles were thought to act as decoys for the immune system by adsorbing specific antiviral antibodies (Dargan and Subak-Sharpe, 1997). This may result in a reduced effectiveness of the initial immune response and in an enhanced likelihood of successful infection by the virus (Subak-Sharpe and Dargan, 1998). Similar observations were made for, e.g., hepatitis B virus, which releases high amounts of non-infectious subviral particles containing the hepatitis B surface antigen (HBsAg) in their membrane upon infection (Chai et al., 2008; Meckes and Raab-Traub, 2011).

First evidence for the occurrence of L-particles *in vivo* was provided by Aleman et al. (2003). They observed that in the nasal mucosa of PrV infected pigs L-particles are present in significant amounts (Aleman et al., 2003). Interestingly, they were able to demonstrate that L-particles were mainly produced by cells that play an important role in the early stages of viral replication (Aleman et al., 2003). This leads to the hypothesis that L-particles fulfill important functions in facilitating an effective infection and also enhancing the viral load early after infection. A second report, highlighting the *in vivo* relevance of L-particles, suggested that these particles are associated with the formation of senile plaques in Alzheimer’s disease patients (Kammerman et al., 2006). Further studies are needed to identify the precise function of L-particles during herpes virus infections.

Notably, L-particle-like non-infectious particles are formed by many other viruses such as the human cytomegalovirus (dense bodies) (Mohammad et al., 2017), hepatitis B virus (subviral VLPs) (Chai et al., 2008) or HIV (membrane delivered microparticles) (Mack et al., 2000). Although their functions are not fully clarified, there is evidence that these particles may transfer viral proteins and miRNAs that are biologically functional, may affect cellular processes and influence the course of infection.

## Possible Use of L-Particles for Vaccination

Classically attenuated viruses created by passaging a virus in cell culture have proven to be extraordinarily effective in protecting against many viral diseases, including smallpox, polio, measles, mumps, and yellow fever (Lauring et al., 2010). Although attenuated viruses are highly immunogenic and induce a potent immune response, their use is limited by safety concerns including the risk to immunosuppressed individuals or evolutionary reversion to high virulence (Bull, 2015). Subunit vaccines consisting of vial components responsible for protective immunity are a much safer alternative to attenuated viruses and were successfully used for vaccination, e.g., against influenza virus (Soema et al., 2015). The major disadvantage of subunit vaccines is their poor immunogenicity (Perrie et al., 2008), wherefore more potent vaccine approaches have been developed. Non-infectious virus-like-particles (VLPs) and virosomes represent a specific class of subunit vaccine that mimic the structure of authentic virus particles (Noad and Roy, 2003). Virosomes are VLPs, consisting of reconstituted viral envelopes with incorporated viral surface antigens, and serve as potent vaccines for inducing virus-neutralizing antibody titers and priming the cellular arm of the immune system (Huckriede et al., 2005). The first successful attempts to immunize people with VLPs were undertaken with serum-purified empty-particles from chronically HBV infected patients and resulted in the development of the first generation vaccine against HBV (Blumberg, 1977). To date, VLPs have been successfully used for the vaccination against numerous viral diseases in pre-clinical studies, clinical trials and a handful of prophylactic VLP-based vaccines, e.g., GlaxoSmithKline’s Engerix (hepatitis B virus) or Cervarix (human papillomavirus) is currently commercialized worldwide (Roldao et al., 2010).

However, there is no effective vaccine against HSV infections to date. Despite successful studies in mice and guinea pigs, a clinical trial with an HSV-2 glycoprotein D-based subunit (gD-2) vaccine has failed (Belshe et al., 2012). With respect to successful vaccination approaches against other enveloped viruses, a virosome-based vaccine might be a safe and potent vaccine candidate to protect against HSV infections. HSV-1 L-particles are virosomes containing, among others, the glycoproteins gB, gD, and gH/gL which are the major stimuli of virus-neutralizing antibodies (Krawczyk et al., 2013; Cairns et al., 2014). Moreover, a genetically engineered HSV-1 expressing a foreign protein [e.g., HIV gp120 as a chimeric fusion protein with an HSV-1 glycoprotein, similar to the construct used in the RV144 HIV vaccine trial (Rerks-Ngarm et al., 2009)], can be expected to produce L-particles containing the recombinant fusion protein, thus enabling engineered HSV-1-derived L-particles to be used as a vaccine to combat diseases other than HSV-1 infections. The most challenging step will be to standardize the separation of HSV-1-derived L-particles from infectious virions. If HSV-1 L-particles could be completely separated from infectious virions or produced in the absence of infectious H-particles, e.g., using a temperature-sensitive mutant of HSV-1 as described by Preston and colleagues (Patent US5384122A) (Preston et al., 1983; Szilagyi and Cunningham, 1991), they might have a considerable advantage over existing vaccine candidates. However, one has to keep in mind that L-particles may have immunosuppressive functions. To overcome this problem, L-particles could be generated by infecting cells with genetically modified viruses lacking proteins involved in immune evasion mechanisms, such as, e.g., ICP0 (Heilingloh et al., 2015). Additionally, potent adjuvants can be used to increase the immunogenicity of L-particles to induce a potent immune response.

## Conclusion

Non-infective microvesicles such as L-particles secreted by cells infected with α-herpesviruses play an important role in the intercellular communication. L-particles were shown to transfer viral and cellular factors to neighboring uninfected cells and thus, facilitate the course of infection. Further studies are needed to clarify the exact mechanisms how viruses can control the viral replication, cellular microenvironment and the hosts’ immune response via microvesicles.

## Author Contributions

CH and AK wrote the manuscript. All authors approved the final version of the manuscript.

## Conflict of Interest Statement

The authors declare that the research was conducted in the absence of any commercial or financial relationships that could be construed as a potential conflict of interest.
